# Experimental and Numerical Assessment of Supporting Road Signs Masts Family for Compliance with the Standard EN 12767

**DOI:** 10.3390/ma14205999

**Published:** 2021-10-12

**Authors:** Michał Stopel, Artur Cichański, Nathalie Yague, Grzegorz Kończalski

**Affiliations:** 1 Faculty of Mechanical Engineering, Bydgoszcz University of Science and Technology, Kaliskiego 7, 85-796 Bydgoszcz, Poland; artur.cichanski@pbs.edu.pl; 2 Transpolis SAS, 620 Route des Fromentaux, FR-01500 Saint-Maurice-de-Remens, France; nathalie.yague@transpolis.fr; 3 Tioman Sp. z o.o. Sp. k., Ostaszewo 57e, 87-148 Łysomice, Poland; gk@tioman.pl

**Keywords:** crash test, LS-Dyna, injury metrics, roadside safety, passive safety, fail-safe

## Abstract

The analysis aimed to assess the passive safety of supporting masts for road signs in accordance with EN 12767. Experimental tests were carried out based on the requirements of the standard for the smallest and the largest constructions within the product family. Numerical models of crash tests were prepared for whole product family using the Finite Element Method in the LS-Dyna environment. Based on the comparison of the experimental tests and the numerical calculations, the usefulness of the numerical model for estimating the actual value of the Acceleration Severity Index (ASI) and the Theoretical Head Impact Velocity (THIV) was assessed. With the use of these relationships the values of ASI and THIV for masts not tested experimentally were estimated. It was confirmed that the analyzed masts met the requirements for the passive safety of structures set out in the standard EN 12767. It was possible since as a result of the impact, the mast column detached from the base, allowing the vehicle to continue moving. The behavior of the masts was primarily influenced by the destruction of the safety connectors. The paper presents the most important elements from the point of view of designing such solutions.

## 1. Introduction

As a result of road accidents, approximately 80,000 people die every year. Speeding and alcohol abuse are considered to be the major cause of road accidents [1]. At present, the issue of the distraction of drivers and pedestrians is becoming more and more significant. [1]. In the case of drivers, such distraction might lead to an unintended change of a motion path. The safety issue is one of the major areas of interest of the automotive industry. Over the years, a number of active safety systems have been developed, at present being a statutorily required standard of automotive accessories in many countries. Previous systems focused on minimization of injuries suffered due to accidents, now their objective is to avoid them [2]. Road accidents with the participation of road infrastructure or the objects in the nearest road vicinity happen not less rarely than those with other road users. In the USA in 2018 only a collision with this type of object was the cause of fatal injuries among 7422 persons [3]. At present, more emphasis is placed on designing road infrastructure which provides maximum safety for drivers and passengers [4] which, from the point of view of the last decade, has contributed to the drop of fatal accidents with the participation of the road infrastructure [3]. In order to maintain the high dynamics of a drop in the number of fatal accidents, alternative solutions for road traffic safety are searched for. The current development trend is to design passively safe devices which, similarly to the systems in vehicles, are dedicated to minimizing injuries suffered by drivers and passengers resulting from accidents caused by unintended movement path change.

Injury intensity coefficients are adopted for the evaluation of the efficiency of the safety systems in the area of road constructions and vehicles, both passive and active. The coefficients may be based on the kinematics of the ATD phantom (anthropometric test device) [5] or of a vehicle [6]. Delta-v parameter which specifies the decrease of vehicle velocity as a result of an impact is the most frequently applied and the simplest in calculation coefficient based on the vehicle kinematics. This simple model does not take into consideration passive and active safety systems vehicles are equipped with. It is widely used for the initial evaluation of supporting structure or VRS (Vehicle Restraint System) behavior [7], however, it is insufficient for the analysis of the impact consequences for vehicle passengers [8]. ASI (acceleration intensity factor) and THIV (theoretical head velocity during an impact) are better indicators, also based upon the kinematics of vehicles. The importance of the ASI parameter has been proved in numerous publications the authors of which strive to prove the dependence between its value and the injuries suffered by accident victims [9,10,11] or try to propose models allowing for estimation of ASI value depending on factors such as the impact conditions or mechanical properties of the tested road infrastructure [12]. Equipping vehicles in an EDR system (Event Data Records) which records data such as vehicle motion, seat belt fastening, or operation of active safety systems in vehicles currently allows for the determination of correlations between injury intensity factors and real injuries suffered as a result of road collisions [6,13]. ASI is a good indicator in the scope of injuries suffered by passengers having their seat belts fastened and for the vehicles equipped with airbags. The value of this parameter at the level of 1.0 corresponds to minor injuries or lack of injuries [9]. The authors of the paper [10] present a correlation between ASI and other parameters which specify the intensity of injuries such as HIC (Head Injury Criterion)—measuring accelerations acting on a passenger’s head and AIS (Abbreviated Injury Scale)—describing injuries of a head and neck area. It has been noted that, despite its limitations, ASI is the best intensity indicator based on the kinematics of vehicles [3]. As a supplement, the authors of the mentioned paper propose to include numeric simulations with ATD phantom into the evaluation of the impact results. The authors [11] noticed that, despite dependencies between ASI value and the risk of injuries, the border between the next two passenger safety levels, according to [14] does not provide a sufficient division between the high and low probability of injuries. In order to separate the acceptable risk of injuries additional consideration of forces acting on the head during the impact is necessary. It was indicated that the THIV parameter is the most appropriate for this purpose. The model assumes free head movement within a conventionally limited space. It indicates the velocity at which the head impacts against the vehicle interior [11,15]. ASI and THIV parameters allow for a much better assessment of the impact results in case when the vehicle passengers have their seat belts fastened. In the case of unfastened seat belts, the parameters present similar efficiency to the delta-v parameter [6]. The phenomenon of the head kinematics is described not only in relation with the velocity at the moment of the impact but also in relation to the velocity after the impact with PHD (post-impact head deceleration) parameter [16] which, similarly to THIV, assumes free passenger movement inside the vehicle and its value, calculated from the moment the head hits the vehicle interior, should not exceed 20 g. Another indicator that describes the intensity of the vehicle passengers’ injuries proposed by the International Organization for Standardization is VPI (Vehicle Pulse Index) parameter [17]. This parameter takes into account the vehicle passenger by adopting the kinematic model mass-spring with a single degree of freedom. The equations for this model include a function considering systems that limit the passenger’s movement. The paper [18] proposes the model for injury evaluation based on the database of passengers’ kinematics, their injuries and the kinematics of the vehicle itself. It is assumed that the model can be applied in real-time in the vehicle providing information to the passengers’ safety systems before and after the collision. Similar research was conducted by Bance et al. [19]. They proposed an efficient calculation method that allows for foreseeing the risk of passengers’ injuries by advanced safety systems incorporated into vehicles.

According to the authors of the paper [20], the creation of the road vicinity which forgives errors of the road users should always be a priority. The authors suggest five steps to be undertaken in order to reach this goal. The final solution, according to the authors, is to introduce vehicle restraint systems VRS. However, this solution should only be adopted in case other solutions are not feasible or would be economically unjustified. The first of the suggested solutions, being at the same time the most obvious one, is to remove an obstacle hazardous to the road users. If a road infrastructure bears such risk and if it cannot be eliminated, it should be re-designed in order to yield due to the impacting vehicle and thus not act as an obstacle along its route. Another step is to remove the risk farther from the road where it is less likely to be hit by the vehicle which denotes from the road. If none of the above can be implemented, the risk-bearing structure should be designed as passively safe, i.e., in such a manner that limits the risk of injuries suffered by the vehicle passengers in accordance with standard regulations [21]. EN 12767-specifies the requirements and method for testing of supporting structures located in the nearest vicinity of the road. This standard, in relation to the target operating conditions, indicates three classes of velocity 50, 70 and 100 km/h, being decisive for the selection of the vehicle velocity during crash tests. Each velocity class has its own assigned category of energy absorption for the tested structure. The standard defines division into three degrees of energy absorption: high–HE, medium–LE and insignificant–NE. Assigning a given object to the applicable energy absorption class is made based upon the evaluation of the vehicle velocity after a collision against the tested mast (delta-v). Apart from referencing the vehicle velocity, EN 12767 standard regulations define five levels of passenger safety, marked from A to E. Assigning to an appropriate safety level is determined based upon values of parameters ASI and THIV specified by the standard (Table 1). The presented table includes part of the standard provisions relating to the velocity class of 100 km/h. Two crash-tests are defined for each velocity class. First at a low velocity-for each class, it equals 35 km/h, and second at high velocity as specified by a given class.

Tests and research described in the literature refer mainly to the road restraint systems VRS based upon the requirements of EN 1317 standard. Ren et al. [22] conducted tests of a road barrier made of S235 steel. In the paper [23] the authors analyzed the barrier reinforced with a compound-foam material. Bruski et.al. in the paper [24] analyzed the vehicle impact against the barrier made of steel ropes, and in the papers [16,25,26] the impact against the barrier made of concrete blocks was analyzed. The number of papers dedicated to the structures compliant with passive safety requirements according to EN 12767 is significantly lower. Baranowski and Damaziak in their research presented the procedure of lighting post-tests in relation to standard requirements and based on this they presented the impact of the selected numerical model parameters on the obtained results [27]. The authors of the papers [28,29] analyzed constructional solutions for lighting masts which, as a consequence, contributed to the decrease of the delta-v parameter. The authors of the paper [30] presented the results of experimental tests for the selected structure from a family of lighting posts made of compound materials and not being a passively safe structure but the structures which absorb the impact energy. Wach analyzed the vehicle impact against the lighting post, gantry and a road sign mast [31]. The objective of these tests was to specify the unreliability of measurement of the injury severity factors such as ASI, THIV and PHD in relation to the requirements imposed for passively safe structures. An issue of gantries made of the light aluminum lattice was discussed by the authors of the paper [32]. The main aspect considered by the authors was providing stability to the structure after the vehicle impact against one of the supports.

At present, numerical analyses are an important factor incorporated into the process of designing safe road structures. Experimental research for the evaluation of the structural behavior in conditions similar to real ones is applied for validation of the developed model [26]. Consequently, the model allows for extended case studies without the requirement of conducting further tests [16]. Büyük et al., in their work analyzed the experiment of a 900 kg vehicle impact against a crash cushion [33]. The tests were mapped numerically, and the validated model was used for the evaluation of the behavior of the designed crash-bag in case of the impact against 1300 kg vehicle. Budzyńsk et al., presented a significant role of numerical simulation application in the process of road infrastructure designing [34]. Correctly verified numerical models may become a source of crucial information on the process of damage, manner of deformation or the influence on the road event participants. However, not in all cases, such analyses must relate to whole structures. Often, the intensity of injuries resulting from the road event is influenced by individual elements and for such elements, detailed numerical models are developed [35,36]. To a vast extent, the analyses of the vehicle impact against road infrastructure are performed with the application of the Finite Element Method. LS-Dyna is the most frequently applied solver for the analysis of this type of phenomenon [3,16,23,24,26,27]. However, one might come across other software dedicated to analyses of road collisions such as V-Sim [37], applying the SDC method (static, dynamic, characteristic-analysis), or with the implemented FEM elements-PC-Crash [31], DyMESH [37].

The objective of the performed analysis was to evaluate whether the masts from the family of passively safe objects meet the requirements of the provisions of EN 12767 standard. The performed analyses assumed the performance of experimental tests for the smallest and the biggest structure, according to procedures as provided for by the standard. In addition, numerical models of crash-tests for all masts included in the above-mentioned product family were developed. Based on experimental and numerical tests, evaluation of suitability for use of the numerical model in predicting a real value of ASI and THIV for the masts not experimentally tested was performed.

## 2. Materials and Methods

### 2.1. Test Object

**Figure 1:**
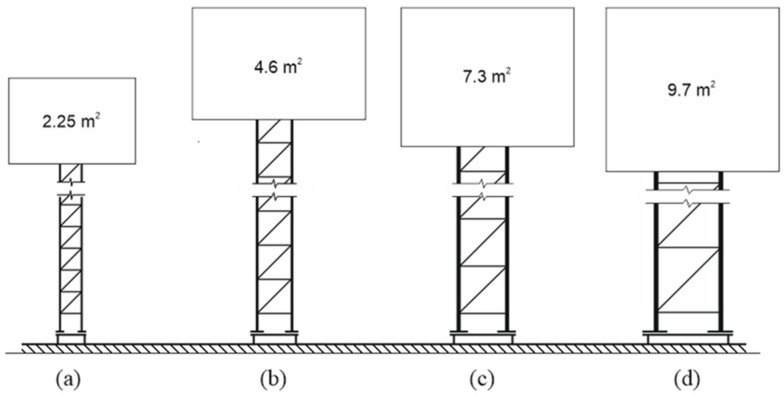
An outline of the product family: (**a**) mast T00, (**b**) mast T01, (**c**) mast T02, (**d**) mast T03.

Tests were conducted for a group of supporting structures of road signs, incorporated in the product family. The product family was understood as a series of objects of the same type, various sizes, made of the same material with the use of the same design and construction method, of the same disconnection or deformation mechanisms during the vehicle impact. The tested product family consisted of four objects analyzed in relation to the requirements imposed on passively safe structures located in the direct road vicinity. Masts marked with T00, T01, T02 and T03 symbols differed mostly by the span of the main rods and their diameters. It corresponded to the ability to support road signs of various surfaces-from 2.25 m^2^ to 9.6 m^2^ (Figure 1).

**Figure 2:**
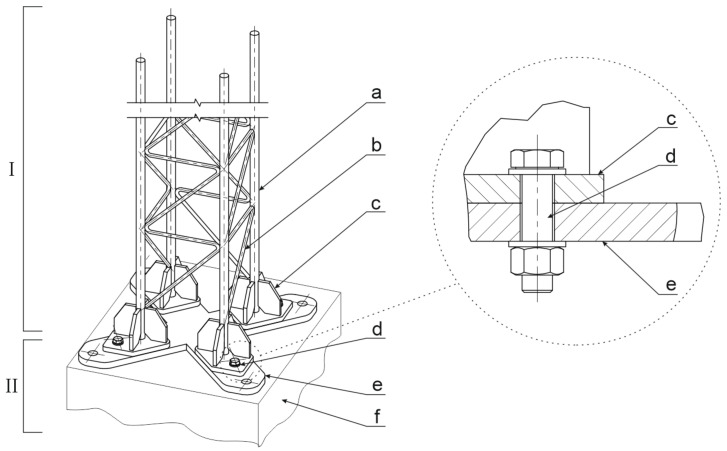
Mast construction.

**Figure 3:**
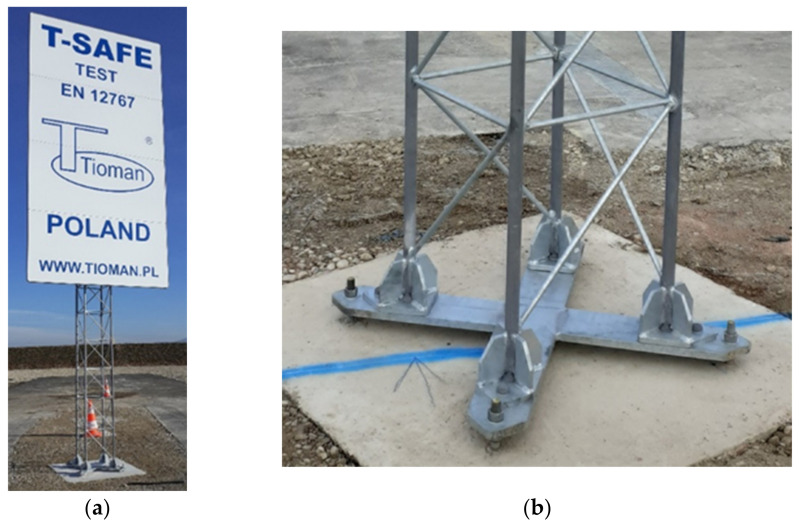
Supporting mast of the road sing.: (**a**) mast T03, (**b**) T03 mast base slab.

In the adopted solution a single mast consisted of a column (Figure 2 mark I) supported on a base (mark II Figure 2). The mast column was made of four main bars with a span on a square plane of a variable side from 200 mm for T00 to 450 mm for T03 (mark a Figure 2). The column was stiffened by the introduction of lattice cross-bars (mark b Figure 2). Each of the four main bars was finished with a pad (mark c Figure 2), connected with the base by screws (mark d Figure 2). The mast base consisted of a base plate (mark e Figure 2), and a foundation (mark f Figure 2), in which it was anchored. The masts of the tested product family met two basic criteria. The first assumed that they would be able to endure operating loads resulting from standard requirements for the signs and supporting structures of the road signs. The second criterion referred to easy mast decomposition as a result of the vehicle impact. In order to match these two conditions, a mechanism for the separation of the columns from its foundation was implemented in the form of screws of 5.8 class acting as a safety joint (mark d Figure 2). It allowed for the separation of the mast column (mark I Figure 2) from its base (mark II Figure 2). Due to the separation of the column from its base, it was possible to obtain a possibly low decrease of the vehicle velocity during the impact which consequently corresponded to a direct decrease of values of the injury severity metrics. The masts T00 and T03 are shown in Figure 3.

All masts were made of S355 JR steel. Each of the mast components was subject to material tests for verification of its properties. As it resulted from the conducted tests, the mechanical properties of the material used for production differed from catalog values (Table 2).

Characteristic dimensions variable for individual product family members were: diameter of main bars, of lattice bars and span of bars (Table 3). With the increase of the diameter of main bars from 16 to 30 mm, their span increased from 200 to 450 mm, resulting in the increase of the masts’ load-bearing value. It allowed the assembly of the signs of a bigger surface from 2.2 m^2^ for mast T00 to 9.7 m^2^ for mast T03.

Experimental tests were performed for masts T00 and T03. Numerical calculations were performed for all masts.

### 2.2. Injury Metrics

EN 12767 standard refers to three parameters of the intensity of injuries. The first is delta-v. The parameter is the basic indicator applied for the evaluation of the road collision results. The parameter describes the value of decrease of the vehicle velocity after its impact against an obstacle. This parameter is the simplest to be determined, however, it is based solely on the vehicle kinematics, thus it does not consider the influence of the passenger restricting systems such as seat belts or airbags. It also does not take into account the time when the vehicle velocity decreases as a result of the manner of deformation of the vehicle crumple zone [13,38].

Contrary to delta-v, the injury parameter *ASI* considers the influence of the passenger restricting systems such as seat belts. *ASI* is determined according to Equation (1),

(1) $$ASI\left(t\right)=\sqrt{{\left(\frac{a_{x}}{\hat{a_{x}}}\right)}^{2}+{\left(\frac{a_{y}}{\hat{a_{y}}}\right)}^{2}+{\left(\frac{a_{z}}{\hat{a_{z}}}\right)}^{2}}$$

where, the numerator of component parts includes the values of the vehicle acceleration in Ox, Oy, Oz axes and its denominator includes threshold values applied according to the standard as: $\hat{a_{x}}=12\;g,\;\hat{a_{y}}=9\;g,\;\hat{\;a_{z}}=10\;g$.

The calculated value of *ASI* is the scalar value, expressed by the Equation (2).

(2) $$ASI=\;\max\;\left[ASI\left(t\right)\right]$$

The third parameter specified by EN 12767 standard is the value of theoretical head impact velocity (*THIV*). It assumes the injuries which are likely to be suffered by the vehicle passenger are directly linked with the passenger’s head impact against the vehicle interior. *THIV* value can be calculated by Equation (3) assuming that the vehicle passenger’s head velocity equals the vehicle velocity in the horizontal plane.

(3) $$THIV=\left[V_{head\;x}{}^{2}\left(T\right)\;+\;V_{head\;y}{}^{2}\left(T\right)\right]$$

where: $V_{head\;x},V_{head\;y}$ are the values of the head velocity in direction longitudinal and lateral to the vehicle axis crossing its center, T is the time when the theoretical passenger head moves by 600 mm in Ox-axis direction or by 300 mm in Oy-axis direction.

### 2.3. Test Setup

**Figure 4:**
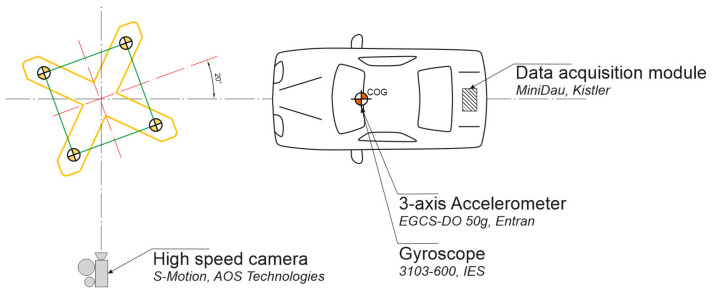
A scheme of mutual location of the supporting mast, the vehicle and measuring instrumentation.

**Figure 5:**
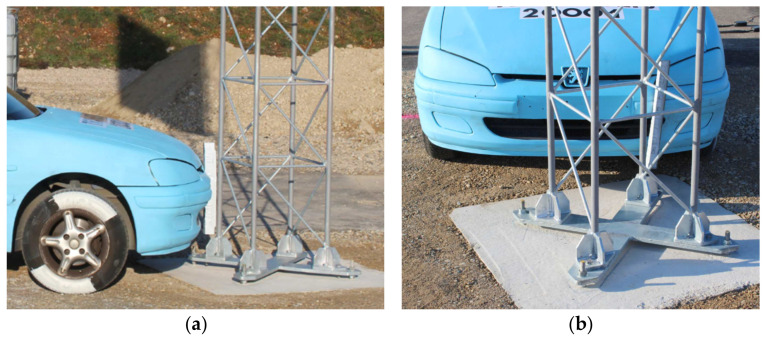
Mutual location of the vehicle and the mast before crash-test: (**a**) side view; (**b**) front view.

Experimental tests were performed on the La Valbonne testing track, located in the test laboratory of Transpolis SAS in Lyon, France. The tests were performed on full-size objects. Test conditions were applied following the requirements of the EN 12767 standard. The tested mast was situated centrally on the route of the moving test vehicle. It was anchored on the concrete foundation poured into stabilized soil. The top surface of the foundation was located on the road level. An angle between the mast symmetry plane and the vehicle movement route was 20° what corresponded to real operating conditions. During experimental tests, Peugeot 106 (1998) was used as a test vehicle. The static mass of the used vehicle was 905 kg. Figure 4 presents a scheme of an experiment, according to the provisions of EN 12767. Figure 5 presents the location of the vehicle in relation to the mast during the performed test experiment.

During the test, the image was recorded with the use of a fast camera (S-Motion, AOS Technologies, Baden Dättwil, Switzerland), triple-axial gyroscope (3103-600, IES Elektronikentwicklung, Braunschwieg, Germany) and triple-axial accelerometer (EGCS-DO 50 g, Entran Devices, Fairfield, NJ, USA). The data measured with the gyroscope and accelerometer were recorded by the data recording module (MiniDau, Kistler, Prague, Czech Republic) and then transferred via wireless network to the PC for processing with the developed calculation software, determining the values of parameters of injury intensity ASI and THIV.

Experimental tests were conducted for mast T00 and T03 with two velocities, resulting from the adopted velocity class of 100 km/h. The applied velocity class directly indicated the velocity at which the high-velocity test was conducted, however, a low-velocity test was performed with the vehicle velocity equal to 35 km/h. The test vehicle was accelerated with the use of a technical vehicle traveling at a controlled velocity. Correct vehicle path was maintained by the guiding rail. 2 m before contacting mast the vehicle was released from the guiding rail.

### 2.4. Numerical Model

The numerical model was developed with the use of LS-PrePost pre-processor and calculations were conducted with the application of LS-Dyna R7.1 solver. Construction of such a complex numerical model was divided into stages which allowed for performing analysis of various phenomena and mechanisms influencing destruction or deformation process as a result of the vehicle impact. From the point of view of compliance with the standard requirements and proper evaluation of the applied constructional solutions, one of the major challenges was the correct modeling of safety joints. Modeled safety joint is a screw with a thread made in burnishing technology. An impact of top layer crumple on macroscopic properties of the whole joint was analyzed in the paper [1]. It showed that the top layer condition does not significantly influence the value of the force necessary for the joint destruction. Moreover, various options of geometrical modeling of its form were analyzed. The use of BEAM, SHELL and SOLID elements was considered. The test results in this area were presented by the authors in the paper [39]. Based upon the performed analyses, the authors decided to model the joint with the application of SOLID type elements. In the same manner, anchors joining the base slab with the foundation were modeled. Another significant phenomenon to be considered was the influence of deformation velocity on change in material properties. The works performed by the authors in this respect are presented in the paper [40]. The conducted tests showed that consideration of a change in material properties is of significant importance for the operation of structures subject to dynamic loads. However, it was also showed that, despite the high velocity of deformation in joints, also great importance was assigned to the process of deformation of the mast column due to the accuracy of modeling of the load transfer mapping from the column to safety joints.

**Figure 6:**
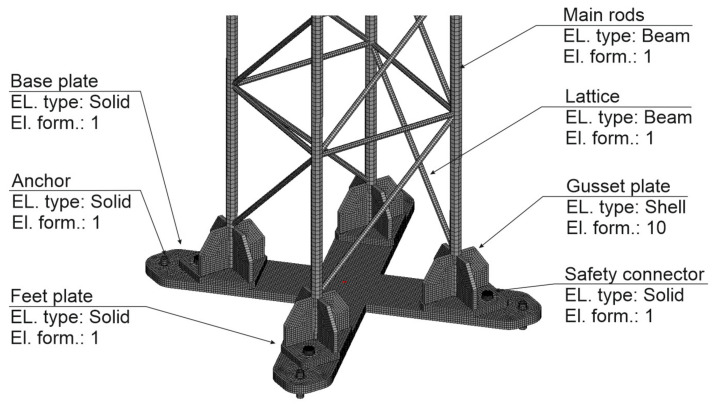
Numerical model of T03 mast.

The numerical model consisted of two objects. The first was the tested supporting mast of the road sign. The numerical model of the T03 mast is graphically presented in Figure 6. Main bars and the mast lattice were modeled with BEAM elements of Hughes-Liu type of circular cross-section with numerical integration with Gaussian quadrature for four integration points. The gusset connecting the mast pad with main rods and the road sign were modeled with the application of SHELL elements of Belytschko-Tsay type with three integration points on their thickness. The base plate, mast pad slabs, anchors and screws working as safety joints were modeled with the use of constant stress SOLID elements. Connection of main bars with lattice bars and gussets was made by merging nodes of components. Gusset joining with the pad was modeled in a similar way. The contacts between mast elements and the vehicle were modeled with contact interface of AUTOMATIC_SURFACE_TO_SURFACE type. In order to model the contact between holes in the base plate and the mast pads and the screws contact elements, ERODING_NODES_TO_SURFACE were applied. This type of contact element allows for the location of new contacting surfaces which appear after the elements prone to limit strain value have been removed from the model. The connection between anchors and the foundation surface was made by taking all freedom degrees off from the nodes located at the anchor base.

For the material described in the numerical model bilinear PLASTIC_KINEMATIC model was applied. The condition of a threshold value of yield deformation was adopted as the failure model. Material constants were adopted based upon test results presented in Table 2. Due to the dynamic nature of loading, it was necessary to fine-tune the values of yield deformation of destruction and yield point. As the safety joints were the critical structural node that was decisive for the behavior of the entire mast, only their material properties were subject to calibration. The process was developed based upon crash-test results conducted with the use of a test platform [7]. The platform was constructed on the basis of a truck vehicle frame which was not subject to destruction during the test. Thus, it was possible to perform numerous crash tests for masts without incurring any necessary costs connected with the destruction of vehicles. The calibration process was based upon a comparison of the selected construction points of the tested masts. Its objective was to obtain the compliance of numerically determining displacement values with the values obtained in experiments. Ultimately, in the case of the tested product family in the numerical model value of strain to failure parameter, FS and Re for joints at the level of 0.37, and 620 MPa were adopted.

**Figure 7:**
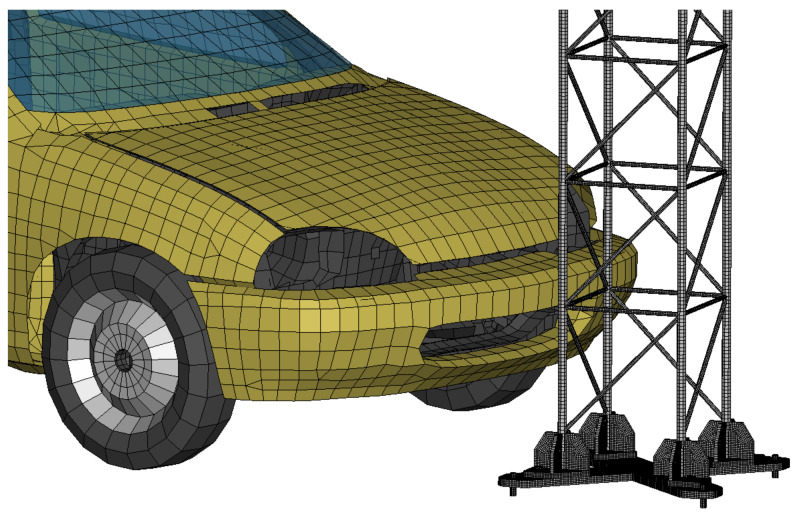
Numerical model of the supporting mast of the road sign with GeoMetro vehicle.

The model of a vehicle Geo Metro (Figure 7) derived from the NCAC database (George Washington University National Crash Analysis Center, Washington, DC, USA) [41] was an important element of the analysis. The vehicle used in the experimental tests weighed 905 kg. The numerical model of the vehicle initially weighed 921 kg, therefore its weight was adjusted to match exactly 905 kg. The size of the test vehicle was 1588 mm wide, 3678 mm long and 1376 mm high, respectively. The numerical model of the vehicle measured 1590 mm, 3750 mm and 1380 mm so used model corresponded to the vehicle used during experimental tests due to its size and total mass. In total, the mast T03 model contained 95,120 finite elements, 131,117 nodes, 8 material models and 13 contact interfaces between components. The calculation time for a single case was 15 h with the application of 11 calculation cores of HP820 operating station, equipped with two Intel Xeon E5-2690 v2 processors.

The vehicle velocity was set with the application of the INITIAL_VELOCITY_GENERATION card giving the nodes incorporated in the wheel structure the rotation velocity about the rotation axis. At the moment of an impact, the vehicle traveled with a linear velocity corresponding to experimental conditions, i.e., 35 km/h or 100 km/h. The supporting mast was fixed in place by removing degrees of freedom from nodes composing the base of anchors connecting the base plate with the foundation. The influence of gravity was taken into account in the calculations.

## 3. Experimental Results

**Figure 8:**
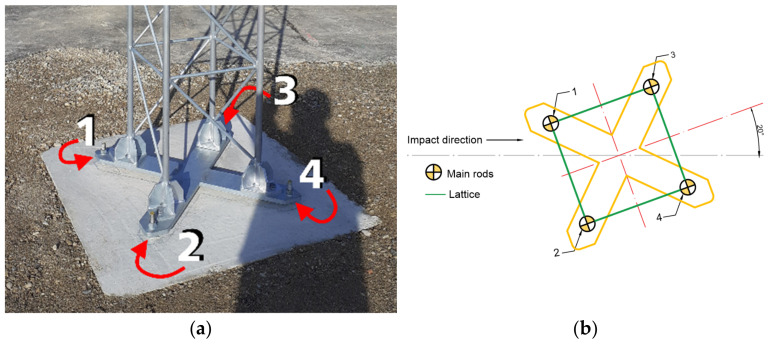
Safety joints breaking pattern: (**a**) mast T03, (**b**) pattern.

**Figure 9:**
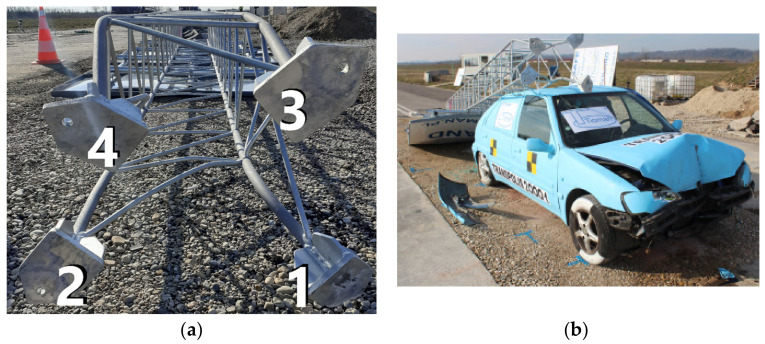
Crash test results: (**a**) main rods deflection, (**b**) vehicle damage.

As a result of an impact, the mast column was deformed which led to the sequential breaking of the joints connecting the pads of main bars with the base plate, starting from the one closest to the impact. The order of breaking the safety joints follows the same pattern in all tested constructions. The order of breaking the bolts is depicted in Figure 8. It was possible since the main mast bars bent heterogeneously, which is shown in Figure 9a. In such a situation, the load in the safety joints is also unevenly distributed, causing the occurrence of stress concentration in the bolt most loaded at the moment. Figure 9b shows damage to the vehicle as a result of hitting the mast column.

**Figure 10:**
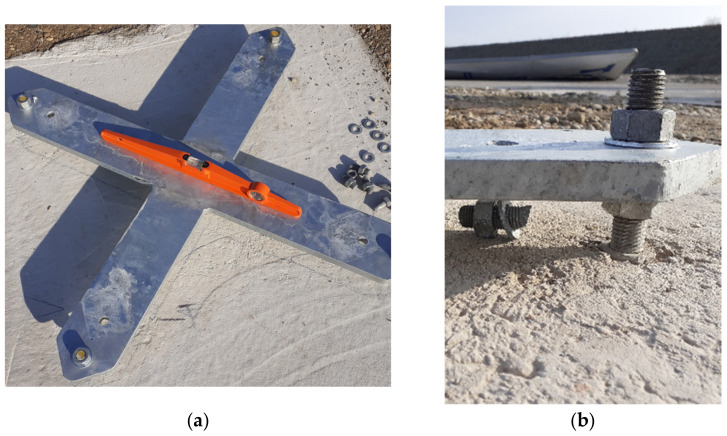
Mast base plate (**a**) and anchors after the experiment (**b**).

It was observed that the concrete foundation did not affect the process. Figure 10 depicts that neither the base plate nor the anchors were deformed.

**Figure 11:**
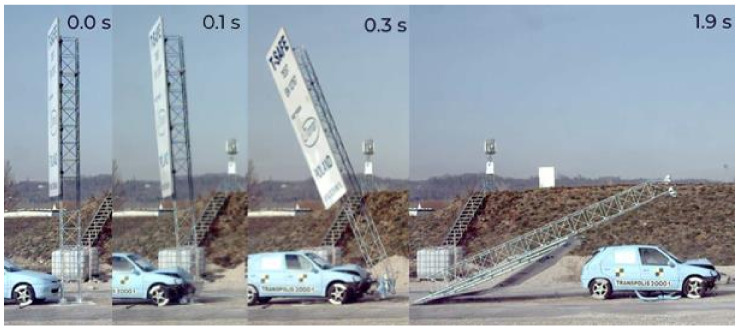
The result of a crash test with T03 mast at 35 km/h velocities.

As the column was separated from the base anchored in the foundation, the mast column, as a result of an impact, rotated around the center of gravity and moved above the traveling vehicle. In the case of the test with lower velocity, the column leaned against the vehicle roof and then slid down without a complete vehicle stoppage. The results of experimental tests were presented in the form of sequential images taken by a fast camera. Figure 11 shows the result of a crash test of a vehicle with a T03 mast at 35 km/h velocities. At the moment of 0.0 s the vehicle contacted the column of the tested mast; at 0.1 s a process of destruction of joints commenced as a result of which at 0.3 s the column was completely separated from its foundation and began to rotate about its center of gravity above the vehicle. At 1.9 s the mast column fell down after the traveling test vehicle. A mechanism of separation of the mast column from its foundation for each of the tested structures was identical.

**Figure 12:**
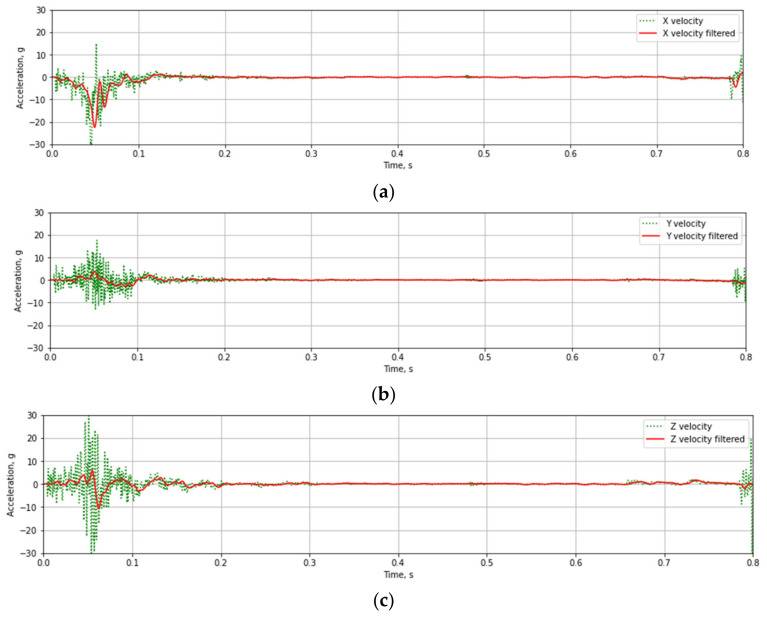
The result of a crash test with T03 mast at 35 km/h velocities. (**a**) velocity in OX-axis, (**b**) velocity in OY-axis, (**c**) velocity in OZ-axis.

**Figure 13:**
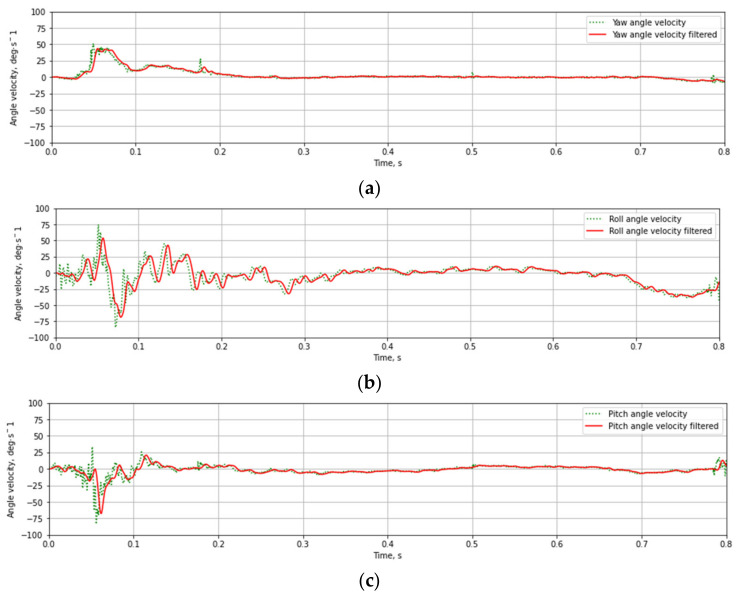
The vehicle acceleration as a result of an impact against T03 mast at 35 km/h: (**a**) rotation in OZ-axis, (**b**) rotation in OX-axis, (**c**) rotation in OY-axis.

The recorded linear acceleration and angular velocity values during testing of T03 mast at 35 km/h velocities are presented in Figure 12 and Figure 13. The data collected during experimental tests were weighed with a digital low-pass Butterworth filter of fourth-order with a 3 dB cut-off frequency equal to 300 Hz (Channel Frequency Class 180) and 100 Hz (CFC 60). Figure 11 and Figure 12 present raw and weighted data.

**Figure 14:**
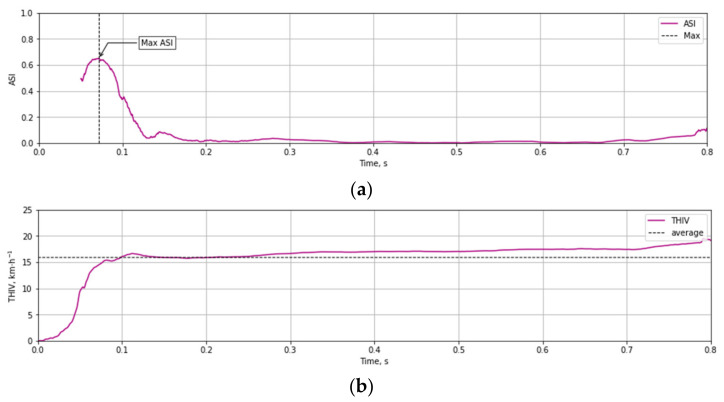
Experimental courses of the values of injury intensity parameters (**a**) ASI, (**b**) THIV as a result of an impact against T03 mast at 35 km/h.

**Figure 15:**
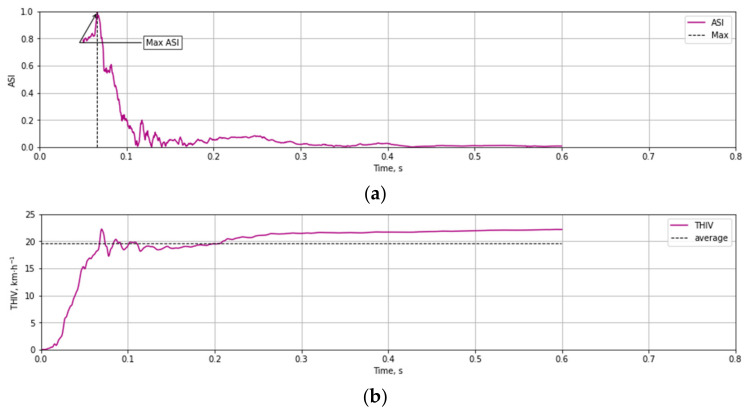
Experimental courses of the values of injury intensity parameters (**a**) ASI, (**b**) THIV as a result of an impact against T03 mast at 100 km/h.

Based on the test data (Figure 12 and Figure 13) and the Equations (1)–(3) time course of ASI (Figure 14a) and THIV (Figure 14b) parameters values were calculated for the test at 35 km/h. Similarly, time courses of ASI and THIV for the test at 100 km/h (Figure 15) were determined. Methodology of calculation of ASI and THIV parameter values based on the data from the accelerometer, with the application of Python 3.0 programming language was presented in the work [42]. The proposed methodology allowed for automation of the process of determination of injury intensity parameters based on raw data directly from measuring instrumentation or being the result of numerical calculations.

Table 4 presents the results of experimental tests for the tested structures of supporting masts T00 and T03.

It can also be noted that for both experimentally tested masts along with increasing impact velocity, the value of injury intensity parameters also increased, whilst both structures meet the requirements of the standard on passive safety. An increase of the value of the evaluated parameters is also achieved along with the increase of main bars span resulting from the geometry of the bigger T03 mast. It should also be noted that, despite the highest main bars span among the hole product family, values of ASI and THIV are also within the limits specified by the standard.

## 4. Numerical Results

Numerical analyses were conducted for all product family members for each test velocity, i.e., 35 and 100 km/h. The paper presents the results of numerical analysis for T03 mast subject to the vehicle impact at the approaching velocity of 35 km/h.

**Figure 16:**
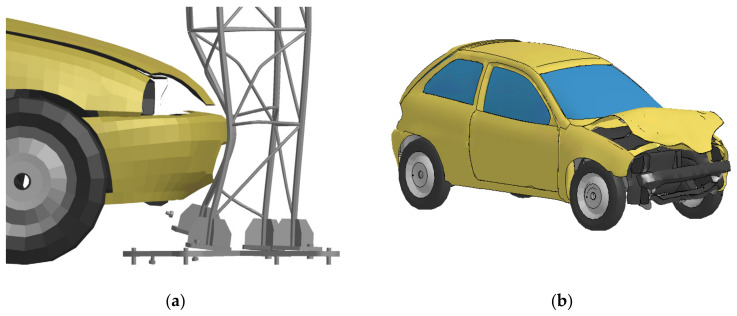
Numerical results Mast T03: (**a**) main bars deflection, (**b**) vehicle damage.

The sequence of damage to the safety joints in the numerical analyzes corresponded to the sequence observed during the experimental tests. The failure of the safety joints occurred as a result of exceeding the set limit value of plastic deformation (0.37). Figure 16a shows the mast T03 column deflection at the moment of breaking of the second safety joint. This is the result of a significant deformation of the main bar mounted on the pad cooperating with this joint. Figure 16b presents the results of the vehicle impact against the T03 mast obtained in numerical analysis.

**Figure 17:**
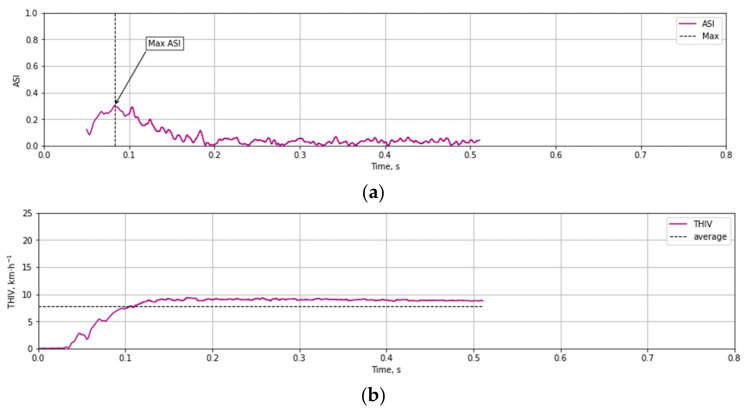
Numerical courses of the values of injury intensity parameters (**a**) ASI, (**b**) THIV as a result of an impact against T03 mast at 35 km/h.

**Figure 18:**
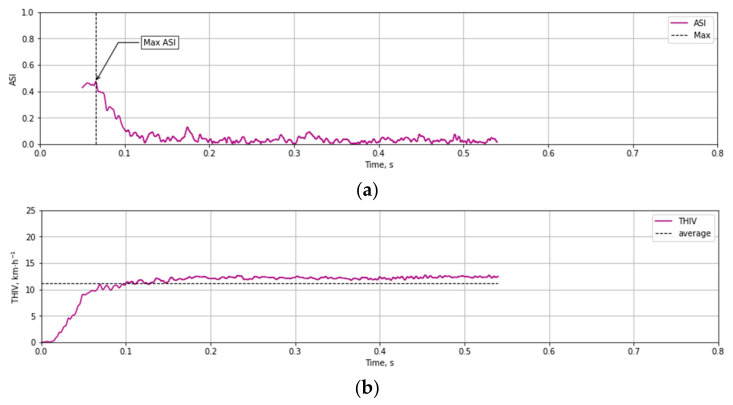
Numerical courses of the values of injury intensity parameters (**a**) ASI, (**b**) THIV as a result of an impact against T03 mast at 100 km/h.

As a result of the performed numerical calculations, based upon registered accelerations and angular velocities, based upon Equations (1)–(3) time courses of values ASI and THIV were determined for all product family members. Figure 17 presents calculation results for T03 mast subject to the vehicle impact at the velocity of 35 km/h, Figure 18 the vehicle velocity is 100 km/h.

The determined values of injury intensity parameters for all product family members, i.e., T00, T01, T02 and T03, are shown in Table 5.

In the case of numerical tests, one can also note the increase of ASI and THIV values along with the increase of the vehicle impact velocity and change in the span of main bars. As it can be seen, the results for T01 and T02 masts are within the area set by two experimentally tested structures.

## 5. Discussion

The main area of interest in the literature discussing the issue of road infrastructure passive safety testing is the road restricting systems that apply safety barriers. Considering the above one can notice a small number of works relating to masts and EN 12767 standard. This gap is filled by the presented tests. One of the current research trends is to develop such methods for the determination of the risk of passenger injuries which might support modern systems of active protection implemented in vehicle accessories. As the requirements provided for by EN 12767 standard are based on well-known ASI and THIV parameters, these parameters were applied for the characterization of the analyzed structures in the work.

The analyzed masts, constituting a family of road sign supporting structures, met the requirements of passive safety of structures specified in EN 12767 standard. As a result of an impact, the mast column detached from its base allowing for further vehicle travel. As a result of an impact, the vehicle decreased its velocity within the limits specified by the standard and the obtained values of ASI and THIV parameters are within an acceptable range. Due to the fact that both the smallest as well as the biggest structure which were experimentally tested met the standard requirements, it was assumed, according to the standard, that the whole family met the requirements in the scope of passive safety. For the class of 100 km/h, both structures proved a high level of passenger safety equal to B in case of low-velocity test, or C in case of high-velocity test.

The standard permits safety level assessment for the whole product family based on experimental tests of the smallest and biggest member of such family, which allows for reduction of costs of a new product launching on the market. The values of ASI and THIV parameters were determined by means of experimental and numerical tests for such structures.

In the case of structure compliant with passive safety requirements, two strength limits can be specified. Minimum structural strength should allow enduring operating loads. Maximum structural strength has a direct influence on the values of injury intensity parameters. The area between the two is usually very narrow. Hence, during simulation tests, one should consider as many phenomena as possible which influence the tested structure. In the case of structures not tested for passive safety, the increased structural strength is an advantage, and its increase is limited solely by economic issues. In the studied case, in relation to standard requirements, it would however lead to the increase of ASI and THIV values and, as a consequence, would contribute to more serious injuries of passengers of the vehicle crashing against the designed masts.

In the case of numerical analysis preparation with an objective to verify the adopted design assumptions and evaluation of the functionality of the developed solution, apart from phenomenological phenomena, consideration of real material properties plays not less significant role. The performed tests showed that the materials used for the construction of supporting masts differed from their catalog values.

In the case of analyzed lattice-based structures, the way how the safety joints break determines the behavior of the mast during the impact. For the mast to be damaged without absorbing energy, the safety joints must break sequentially. The order in which the safety joints break changes the mast stiffness during impact. The proposed numerical model reflects well the order of safety joints breaking and the changes in the stiffness of the mast legs during experimental tests.

## Figures and Tables

**Table 1:** Levels of the safety of the vehicle passengers for the velocity class 100 km∙h^−1^ according to EN 12767 [21].

Index	Test Velocity km/h	Passengers’ Safety Level				
		A	B	C	D	E
**ASI**	35	No test reqired	0.6	1	1	1
	100		0.6	1	1.2	1.4
**THIV, km/h**	35		11	27	27	27
	100		11	27	33	44

**Table 2:** Material test results.

Component	Yield Strength, Re, MPa		Ultimate Tensile Strength, Rm, Mpa		Elongation, A %	
	Nominal	Test	Nominal	Test	Nominal	Test
Main bars	355	450	470–630	536	22	34
Lattice bars		590		657		19
Base slab, pad		448		495		32
Joint, screw 5.8	400	581.1	500	673.1	-	-

**Table 3:** Main geometric properties of masts.

	T00	T01	T02	T03
Surface of the sign, m^2^	2.25	4.6	7.3	9.7
The span of main bars, mm	200	250	350	450
Diameter of main bars, mm	16	20	24	30
Diameter of grating bars, mm	8	10	12	14
Joint, screws	M10	M12	M16	M16

**Table 4:** Experimental tests results.

Km/h	Mast	ASI	THIV	Delta-v
35	T00	0.325	7.366	17.0
	T03	0.65	15.891	n.d.
100	T00	0.348	6.142	94.6
	T03	0.994	19.551	77.9

**Table 5:** The table of numerical test results.

km/h	Mast	ASI	THIV	Delta-v
35	T00	0.136	3.536	32.0
	T01	0.181	3.485	31.0
	T02	0.292	6.512	27.5
	T03	0.301	7.717	26.0
100	T00	0.215	6.075	96.0
	T01	0.294	7.515	91.5
	T02	0.455	10.988	89.0
	T03	0.474	11.089	87.5

## Data Availability

Data sharing is not applicable.
